# Long non-coding RNA-based signature for predicting prognosis of hepatocellular carcinoma

**DOI:** 10.1080/21655979.2021.1878763

**Published:** 2021-02-23

**Authors:** Jie Cao, Lili Wu, Xin Lei, Keqing Shi, Liang Shi, Yifen Shi

**Affiliations:** a Translational Medicine Laboratory, The First College of Wenzhou Medical University, Wenzhou, China; b Department of Clinical Transfusion, The Eighth Affiliated Hospital, Sun Yat-sen University, Shenzhen, China; c Translational Medicine Laboratory, The First Affiliated Hospital of Wenzhou Medical University, Wenzhou, China; d Department of Hematology, The First Affiliated Hospital of Wenzhou Medical University, Wenzhou, China

**Keywords:** Long non-coding RNAs, hepatocellular carcinoma, prognosis, The Cancer Genome Atlas, overall survival

## Abstract

Long non-coding RNAs (lncRNAs), as one common type of non-coding RNAs, play a critical role in the tumorigenesis and development of hepatocellular carcinoma (HCC). In the current study, we aimed to assess the correlation between lncRNAs expression levels and prognosis of HCC patients. A lncRNA-based signature was also developed to predict the prognosis of HCC in this work. The lncRNAs expression profiles in tissues of tumor and para-carcinoma were obtained from The Cancer Genome Atlas (TCGA) database. The lncRNA-based prognostic model was established by least absolute shrinkage and selection operator (LASSO). The multivariate Cox-regression analysis was applied to identify the independent risk factors and subsequently developed a prognostic nomogram. Based on the co-expression analyses, we identiﬁed the lncRNA-related mRNAs and performed the biological function analysis. Between HCC and para-carcinoma tissues, 220 differentially expressed lncRNAs were filtered. Among these lncRNAs, 19 lncRNAs were identified as prognostic factors and were used to build a prognostic signature of overall survival (OS). Furthermore, a nomogram with high performance for predicting the OS of HCC patients (C-index: 0.779) by combining the 19-lncRNA signature (P < 0.001) and clinicopathologic factors including HBV (P = 0.005) and stage (P =0.017) was established. Functional enrichment analysis revealed that 19 lncRNAs had potential effects on tumor cell proliferation in HCC. In summary, we established a 19-lncRNA signature to predict the prognosis of HCC patients, which may perform a crucial role in guiding the management of HCC.

## Graphical abstract

**Figure:**
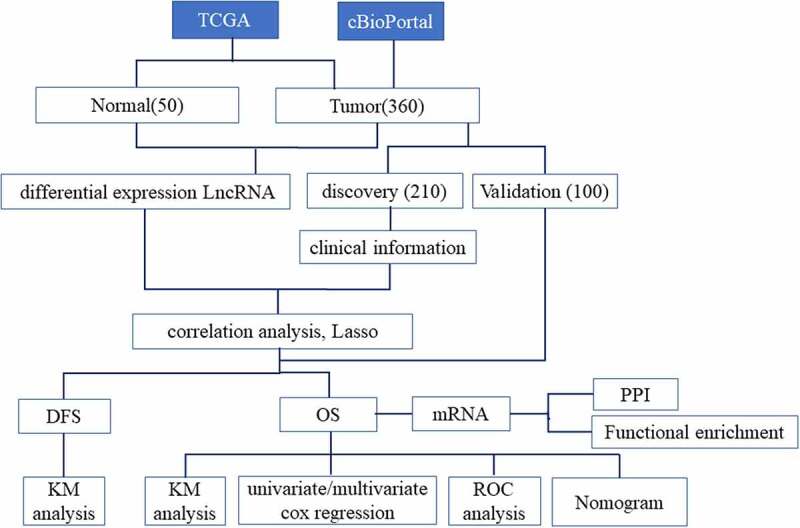

## Introduction

Liver cancer is a highly aggressive malignant disease with increasing incidences and mortalities [1]. Hepatocellular carcinoma (HCC), the fourth primary cause of cancer-related deaths worldwide, is the most important pathological classification of liver cancer [2]. To date, various methods have been used to treat HCC, such as liver resection, percutaneous ablation, liver transplantation, gene therapy, immunotherapy and genomic-targeted therapy. However, the 5-year survival rate of patients with HCC is still very low. This is mainly due to the intra-heterogeneity that cells in the tumor microenvironment often harbor distinct molecular signatures [3]. Hence, even HCC patients with uniform tumor stage are different in treatment sensitivity levels and prognosis. Recently, with the widespread application of the technology of sequencing [4], many researches have revealed that molecular pathological subtyping and driving gene mutations significantly affected the prognosis of HCC patients [5]. However, the underlying molecular pathological mechanisms of the progression of HCC remain unclear [6]. Therefore, a valid molecular-based signature model is urgently needed to provide a precise prediction for the prognosis of HCC.

Long non-coding RNA (lncRNA) is a crucial member of non-coding RNAs without protein-coding functions and has greater than 200 nucleotides in length. LncRNA is reported to play a vital role in regulating various biological functions including apoptosis, cell proliferation, tumorigenesis, and metabolism [7–9]. Accumulated data have reported the potential role in predicting the prognosis of cancer patients [10,11]. For instance, some studies have indicated that lncRNA affected the prognosis of liver cancer. The lncRNA TPTEP1 restrained HCC cells invasion and proliferation by affecting IL-6/STAT3. The finding suggested that highly expressed TPTEP1 promotes the prognosis of HCC [12]. Based on the PDK1/AKT/caspase 3 pathway, lncRNA PDPK2P had interaction with PDK1 to promote HCC development, indicating it can act as a target gene for the therapy and prognosis of HCC [13]. MiR-214 was sponged by the MIAT to accelerate the invasion and proliferation of HCC cells. The lncRNA MIAT/miR-214 axis provided a new insight for poor HCC prognosis [14]. LncRNA MCM3AP-AS1 had an oncogenic function by targeting miR-194-5p to promote the expression of FOXA1, which showed poor prognosis in HCC patients [15]. These evidence suggested that lncRNAs were closely related to the prognosis of HCC.

In this research, we studied the lncRNA expression profile in HCC and para-carcinoma tissue to screen out differentially expressed lncRNAs. Based on the differentially expressed lncRNA, the lncRNA-based signature was established for predicting the HCC patient prognosis. Moreover, two models, single model contained the clinicopathological information and the combined model contained the clinicopathological information and prognostic score, were constructed to prove the significance of lncRNA-based prognostic signature. A nomogram combined the lncRNA signature and clinicopathologic factors was used to predict the survival rates of HCC, guiding individualized clinical treatment for HCC patients.

## Methods

### Patients and database

LncRNA expression profiles of 360 HCC samples and 50 para-carcinoma samples were obtained from The Cancer Genome Atlas (TCGA) database [16], and corresponding clinical data of 360 HCC samples were obtained from the TCGA database (https://portal.gdc.cancer.gov/) and cBioPortal database (https://www.cbioportal.org/) [17]. (Table 1) showed the clinicopathological characteristics of 360 patients. Of the 360 cases, we filtered 310 cases, which included the information of OS and DFS. The 310 cases were randomly separated into training cohort (n = 210) and validation cohort (n = 100). There were no significant differences in the sex (p = 0.710) and age (p = 0.865) distribution between the training cohort and validation cohort.

**Table 1.** Clinicopathological characteristics of 360 hepatocellular carcinoma patients

Parameter	N
Sex	
Male	242
Female	118
Age	
<=60	171
>60	189
Family history	
Yes	112
No	200
Alcohol	
Yes	114
No	228
HBV	
Yes	100
No	242
HCV	
Yes	53
No	289
AFP	
<=25	160
>25	113
Grade	
I	54
II	171
III	118
IV	12
Stage	
T1	176
T2	90
T3	77
T4	13

Abbreviation: HBV, hepatitis B virus; HCV, hepatitis C virus; AFP, alpha-fetoprotein.

### Procedure

Firstly, differentially expressed lncRNAs and mRNA between HCC and para-carcinoma were screened by the Deseq2 package in R [18]. Heatmap and Volcano plot of differentially expressed lncRNAs were also created. Then, differentially expressed genes were served as candidate variables and used for the survival analysis.

Secondly, LncRNAs that had a significant correlation with OS and DFS were screened by univariate cox analysis. The p-value <0.05 was defined as statistical significance. The prognostic lncRNAs were entered into lasso analysis which was established by the glmnet and survival package. The lncRNA-based prognosis score was the sum–product of gene expression and their corresponding lambda-value as follows:

Prognostic score = expr-gene1 × λgene1 + expr-gene2 × λgene2 + … + expr-genen × λgenen Expr-gene: expression values of genes. λgene: corresponding lambda-values of genes. According to the prognostic score, we obtained optimal cutoff values using the ‘survminer’ package in R. All HCC patients were ultimately separated into high or low-risk groups by optimal cutoff values.

In order to detect whether the risk score was the independent biomarker of prognosis, we proceed a multivariate analysis for the clinicopathological characteristics and prognostic score. The Receiver-Operator Characteristic (ROC) analysis was applied, via the ‘timeROC’ in R using the default parameters to demonstrate the sensitivity/specificity of lncRNA-based prognostic signature in prognosis prediction [19]. Based on the ROC curve, the area under the curve (AUC) was obtained. The drawing of ROC results of the lncRNA-based prognostic score was performed by the ‘ggplot2’ package.

Then, the nomogram was constructed by variables with P-values (<0.05) in the multivariate analysis, which included the lncRNA-based prognostic signature and clinicopathologic factors. The concordance index (c-index) was displayed to measure the predictive capability of nomogram by using the ‘rms’ package in R software.

The protein–protein interaction network (PPI) was obtained by the STRING database (http://string-db.org) [20]. The interaction with a combined score >0.4 (medium confidence) was thought statistically significant. The Cytoscape [21], acting as an information biology software, was applied to analyze and visualize molecule interaction network. The well-connected regions in the molecule interaction network were selected by plug-in MCODE of Cytoscape. Metascape (https://metascape.org/gp/index.html) was used to analysis the gene enrichment of KEGG, cellular components (CC), biological processes (BP) and molecular function (MF) [22].

### Statistical analysis

The OS was defined as the time between the date of first diagnosis and death as a result of any cause. The DFS was defined as the time between the date of the first surgery and first recurrence or death. Survival analysis of OS and DFS was shown by the Kaplan Meier in R software. A p-value <0.05 was acted as the statistical significance of differences. The statistical analysis was reported by 95% confidence intervals (CIs). We used R version 3.6.0 software and SPSS version 25.0 Software.

## Results

In the present research, we aimed to build a lncRNA-based signature to predict the prognosis for patients with HCC. We explored the lncRNA expression levels in HCC and para-carcinoma tissue to filter out differentially expressed lncRNAs and these differentially expressed lncRNAs were applied to establish a prognostic model. In addition, based on the clinicopathologic information and lncRNA-based signature, a nomogram was constructed for predicting the patient survival rates. Functional enrichment analysis was also proceeded to explore the underlying functions of the alternative lncRNAs.

### Differentially expressed lncRNAs in HCC and para-carcinoma tissues

**Figure 1.**
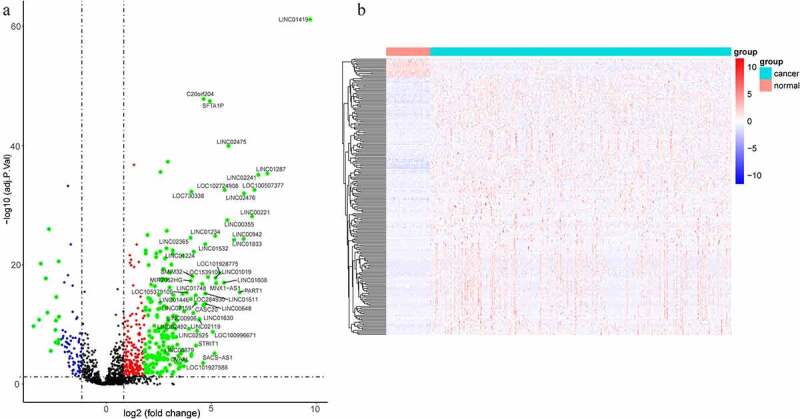
LncRNAs profiling of tumor and para-carcinoma tissues. (a) Heat-map showing profiles of lncRNAs in tumor and para-carcinoma tissues. red represented that the expression of lncRNAs is relative upregulation, blue represented that the expression levels of lncRNAs is relative downregulation. (b) Volcano plots of differential expression lncRNAs in HCC and para-carcinoma tissues. the red points represented up-regulated lncRNAs (1< log2-fold change< 2, P < 0.05). the green points represented down-regulated lncRNAs (−2< log2-fold change< -1, P < 0.05). the blue points represented up-regulated and down-regulated lncRNAs (| log2-fold change | >2, P < 0.05). each black point represented genes with a non-significant difference

The lncRNA expression levels in tumor and para-carcinoma tissues were obtained from the TCGA database. Results found that the lncRNA expression profile was different between tumor and para-carcinoma tissues (Figure 1). When the different expression of the same lncRNA in tumor and para-carcinoma tissues was more than 2-folds, the difference of the expression was regarded as significant. A total of 220 lncRNAs with significantly different expression levels were identified, including 205 upregulated and 15 down-regulated genes.

### LncRNA-based signature predicting OS

**Figure 2.**
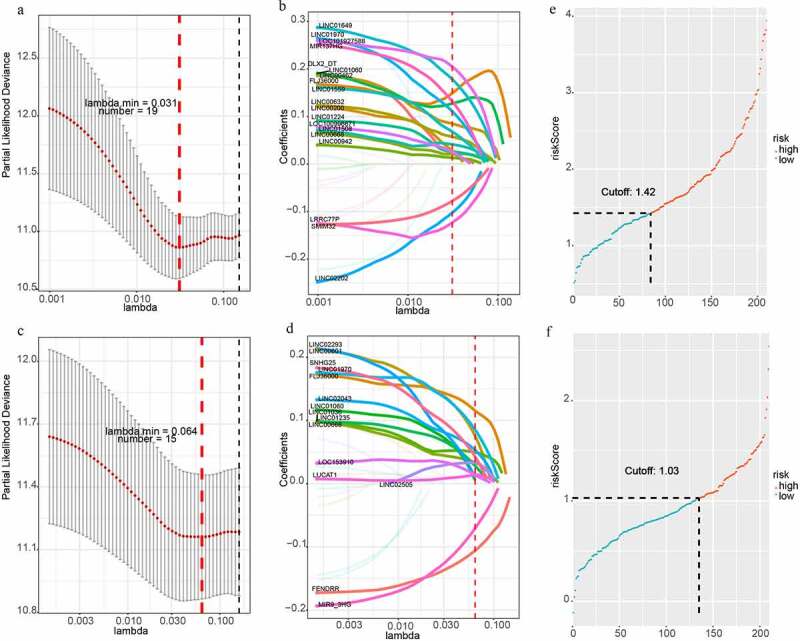
LncRNA-based prognostic signature of HCC. (a)(b) LASSO cox analysis showed Nineteen genes were associated with OS in the discovery group. (c)(d) LASSO cox analysis showed Fifteen genes were associated with DFS in the validation group. (e)Prognostic scores distribution for the discovery group in OS. (f) Prognostic scores distribution for the validation group in DFS

To build two more precise prognostic models, 220 candidate lncRNAs were used to perform the univariate Cox-PH regression. The expression of 32 lncRNAs was related to OS, among which 29 lncRNAs showed a positive correlation and 3 lncRNAs had a negative correlation. In addition, the expression of 25 lncRNAs was correlated with and DFS, among which 23 lncRNAs showed a positive correlation and 2 lncRNAs had a negative correlation. Next, LASSO analysis was applied to filter genes with the optimal prognostic value. Finally, 19 lncRNAs and 15 lncRNAs were identified to be associated with OS and DFS, respectively (Figure 2a-d). Therefore, these two lncRNA-based prognostic signature could be used to predict OS and DFS in HCC patients.

**Figure 3.**
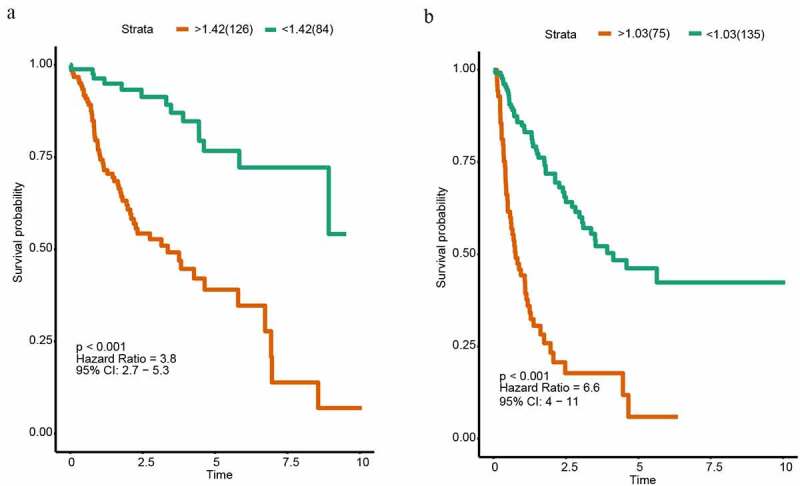
Kaplan-Meier analysis of HCC in the discovery group. (a) Kaplan–Meier curve of OS in the discovery group. (b) Kaplan–Meier curve of DFS in the discovery group

For each HCC patient, the lncRNA-based risk score was computed (Figures 2e and 2f). We selected the best cutoff values by the survminer package in R and divided patients into two subgroups (high risk and low-risk group) based on the prognostic scores. The cutoff value of OS and DFS were 1.424 and 1.030. Patients belonging to the high-risk subgroup had shorter OS (HR = 3.8, P < 0.001, Figure 3a) and DFS (HR = 6.6, P < 0.001, Figure 3b). Subsequently, we used another 100 samples as a validation group. According to the signature, the risk scores of all HCC patient were calculated. The patients were also separated into high-risk and low-risk group. The cutoff value of OS and DFS were 1.746 and 1.007. Kaplan Meier analyses indicated that the high-risk group had the significantly shorter OS (HR = 1.7, P = 0.011, Supplementary Figure 1A). However, Kaplan Meier analyses demonstrated that the lncRNA-based prognosis signature for DFS was not significant in the validation group (HR = 2.5, P = 0.064, Supplementary Figure 1B).

### lncRNA-based prognostic score and applications

**Figure 4.**
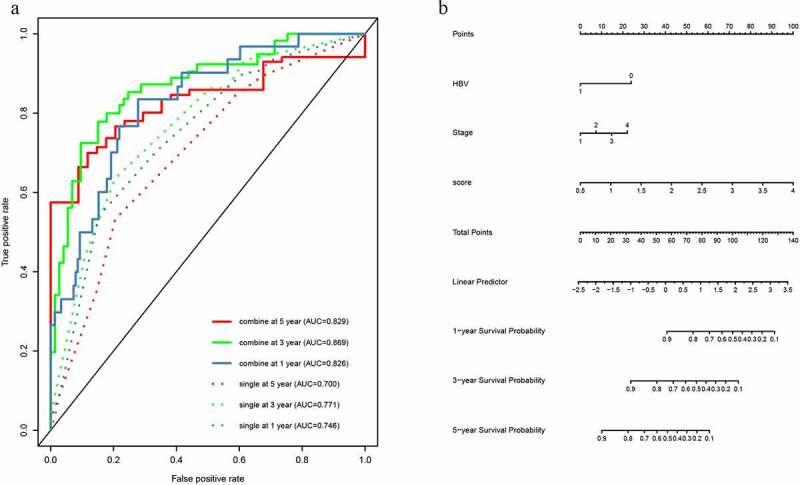
The time-dependent ROC analysis and nomogram for the lncRNA-based prognostic signature. (a)The time-dependent ROC analysis of the combined model included the clinicopathological information and the single model included clinicopathological information. (b) A nomogram to predict the probability of OS in hepatocellular carcinoma

According to the data containing both lncRNA-based signature and clinical information, univariable and multivariable Cox analyses were applied to determine the prognosis factors for OS of HCC patients. Univariate Cox analysis showed that age (P = 0.046), hepatitis B virus (HBV) (P < 0.001) and stage (P < 0.001) were significant clinicopathological risk factors related with HCC prognosis. The multivariate Cox regression analysis revealed that factors of the score of 19-lncRNA signature (P < 0.001), HBV (P = 0.005) and stage (P = 0.017) were associated with OS of HCC prognosis (Table 2). Subsequently, we constructed two multi-variate models to prove the significance of lncRNA-based prognostic score in predicting survival. The single model only contained the significant clinicopathological information, and the combined model contained the significant clinicopathological information and prognostic score. The ROC analysis and AUC calculations were performed to assess both models. For 1-year, 3-year and 5-year survival times, the AUC value of the combined model was 0.826 (95% CI 0.761–0.891), 0.869 (95% CI 0.815–0.923) and 0.829 (95% CI 0.752–0.907), respectively; while AUC of this single model was 0.746 (95% CI 0.668–0.823), 0.771 (95% CI 0.705–0.837), 0.700 (95% CI 0.609–0.791) (Figure 4a). The calibration curves also showed two models performing well in predicting the 1-year, 3-year and 5-year prognosis of HCC patients (Supplementary Figure 2). The predictive ability of the combined model which contained lncRNA-based prognostic score was better (Supplementary Table 1), indicating that the lncRNA-based prognostic signature served as a significant and crucial prognosis factor.

**Table 2.** Univariate and multivariate Cox PH regression in survival analysis

	Univariate			Multivariate		
Parameter	HR	95%CI	P value	HR	95%CI	P value
Sex	0.700	(0.440,1.114)	0.132			
Age	1.019	(1.000,1.038)	0.046	1.008	(0.988,1.029)	0.418
Family history 1.290		(0.808,2.061)	0.286			
Alcohol	1.267	(0.790,2.034)	0.326			
HBV	0.275	(0.145,0.524)	<0.001	0.377	(0.191,0.744)	0.005
HCV	1.036	(0.474,2.267)	0.929			
AFP	1.000	(1.000,1.000)	0.487			
Grade	1.246	(0.912,1.703)	0.167			
Stage	1.716	(1.333,2.209)	<0.001	1.399	(1.063,1.841)	0.017
Prognostic score 3.788		(2.735,5.246)	<0.001	3.433	(2.436,4.838)	<0.001

According to the combined model contained crucial clinicopathological information and the lncRNA-based prognostic score, we built a nomogram (Figure 4b). The clinicopathological information contained HBV and tumor stage. The concordance index (C-index) for the prediction of OS was 0.779.

### Functional characteristics of 19 prognostic lncRNAs

We studied the co-expressed genes of 19 lncRNAs by computing Pearson correlations between the 19-lncRNA signature and 1662 differentially protein-coding genes in the TCGA data portal to explore the latent effect of the 19 lncRNAs in HCC. The filtering condition of differentially protein-coding genes was positively correlated with at least one lncRNA served as (Pearson coefficient >0.4, P < 0.01). A total of 393 genes were filtered for subsequent analysis (Supplementary Figure 3A). To obtain the important module, the Cytoscape software and the STRING database were used to analyze the 393 co-expressed genes. The protein–protein interaction (PPI) network of coexpressed genes was built and the most important module including 102 genes was gained by Cytoscape (Supplementary Figure 3B and 3C). Functional enrichment analyses of module genes were displayed by metascape. The research showed that the 102 module genes were significantly enriched in the cell proliferation-related pathways, such as cell division, spindle, regulation of cell cycle process and cell cycle, etc. (Supplementary Figure 3D). It revealed that these 19 lncRNAs might have important effects in regulating tumor cell proliferation.

## Discussion

Liver cancer was a common and lethal human malignancy in the world. Genetic alterations and prognosis of liver cancer have been broadly and deeply explored [23–25]. In the last few years, many genetic markers have been shown to play a crucial part in prognostic and were broadly used in prognostic signatures [26]. As we all know, the prognostic signatures predicted the HCC patient prognosis from different aspects. In our research, a prognostic signature was established including 19 lncRNAs to predict the HCC patient prognosis. Based on the lncRNA prognostic signature, we investigated HCC from a novel perspective.

In this study, we obtained differentially expressed lncRNAs in tumor and para-carcinoma tissues from the TCGA database. We regarded these prognosis-related lncRNAs as variables to build the HCC prognostic signature. According to the clinicopathological information and prognostic signature, two multi-variate models were constructed to perform the significance of lncRNA-based prognostic signature in predicting survival, and the prediction ability of the model combining lncRNA and clinicopathological information was better. Based on the combined model including clinicopathological information and lncRNA-based prognostic score, a nomogram was built. Moreover, through analyzing the correlation between diﬀerential expression mRNAs and lncRNAs, the potential biological functions of the 19 lncRNAs were explored in HCC.

Among the 19 lncRNAs in the current study, three of them, including SMIM32, LINC02202, and LRRC77P, acted as protective factors, while the other 16 lncRNAs, including FLJ36000, LINC01649, LINC00462, LINC01060, MIR137HG, DLX2_DT, LOC101927588, LINC01508, LINC01224, LINC00668, LINC01559, LINC00632, LINC00200, LINC00942, and LOC100996671 were risk factors. Among these lncRNAs, LINC00462, LINC01224, LINC00668, and LINC01559 have been demonstrated to affect the prognosis and pathogenesis of cancer. LINC00462 acted as the target gene of miR-665. The overexpression of LINC00462 improved TGFBR1 and TGFBR2 expression levels to activate the SMAD2/3 signaling pathway in pancreatic cancer [27]. The knockdown of LINC00462 in HCC cells contributed to a low aggressive oncogenic phenotype, and the reduction of LINC00462 resulted in the inhibition of the PI3K/AKT signaling pathway [28]. The silencing of LINC01224 could down-regulate the expression levels of CHEK1 via competitively combining with miR-330-5p to restrain HCC progression. The result showed a new pathology molecular mechanism for HCC patients [29].

Zhao et al. displayed that the expression of LINC00668 was related with age, clinical stage, T stage, pathological diﬀerentiation degree, and cervical lymph node metastasis, and proved that knockdown of LINC00668 could suppress the migration, invasion and proliferation ability of laryngeal squamous cell carcinoma [30]. LINC01559 had an interaction with YAP, which might inhibit YAP phosphonation and promote YAP transcription. The finding revealed that YAP serves as the target in LINC01559-regulated pancreatic cancer (PC) development [31].

Compared with other prognostic signatures, our signature had obvious advantages. Firstly, we used the TCGA database which contains a wide variety of data types in varieties of cancers, the sample size of the RNA-seq data was big, the clinical information was also available, and there was complete survival information of liver cancer patients.

Furthermore, both univariate and multivariate Cox analyses indicated that the 19-lncRNA signature had a good predictive ability for prognosis in patients with HCC. According to the area under the ROC curves, the specificity and sensitivity of signature were assessed (AUC >0.7 shows that the signature has a good sensitivity). In addition, we identified an inclusive nomogram that contained a 19-lncRNA-based signature and two clinicopathological information of HBV and tumor stage, which made use of both genomic data and clinical information to enhance the predictive accuracy.

We also acknowledged that the current study still has limitations. One of the important limitations of our study was that the validation of the results in other data sets was lacking. More studies including larger data should be carried out to prove the stability and veracity of the established prognostic models.

## Conclusion

In summary, we identified the lncRNA signatures for HCC prognosis, showing the potential clinical application value of lncRNAs which may have an important influence on the HCC. Further functional studies are required to expound the underlying molecular mechanisms of these lncRNAs in HCC.

## Supplementary Material

Supplemental Material

## Data Availability

The datasets generated and analyzed during the current study are obtained from TCGA (https://portal.gdc.cancer.gov) and cBioPortal database (https://www.cbioportal.org/).

## Highlights

1. A 19-lncRNA signature was established to predict the HCC patient prognosis.

2. The multivariate Cox-regression analysis was applied to identify the independent risk factors.

3. Functional enrichment analysis revealed potential effects of 19 lncRNAs in HCC.
