# Supplementary material for: Long non-coding RNA-based signature for predicting prognosis of hepatocellular carcinoma
Source: Bioengineered. 2021 Feb 23;12(1):673–81. doi: 10.1080/21655979.2021.1878763 (PMC8291889; doi:10.1080/21655979.2021.1878763)
Supplement: Supplemental Material [file KBIE_A_1878763_SM2204.zip › supplement/Table S1.docx]

| **Table S1. The p values of the combine model and the single model** | | | |
| --- | --- | --- | --- |
|  | **year** | | |
| **Parameter** | **1** | **3** | **5** |
| P value | 0.070 | 0.005 | 0.001 |
