# Supplementary figures and images for: Long non-coding RNA-based signature for predicting prognosis of hepatocellular carcinoma
Source: Bioengineered. 2021 Feb 23;12(1):673–81. doi: 10.1080/21655979.2021.1878763 (PMC8291889; doi:10.1080/21655979.2021.1878763)

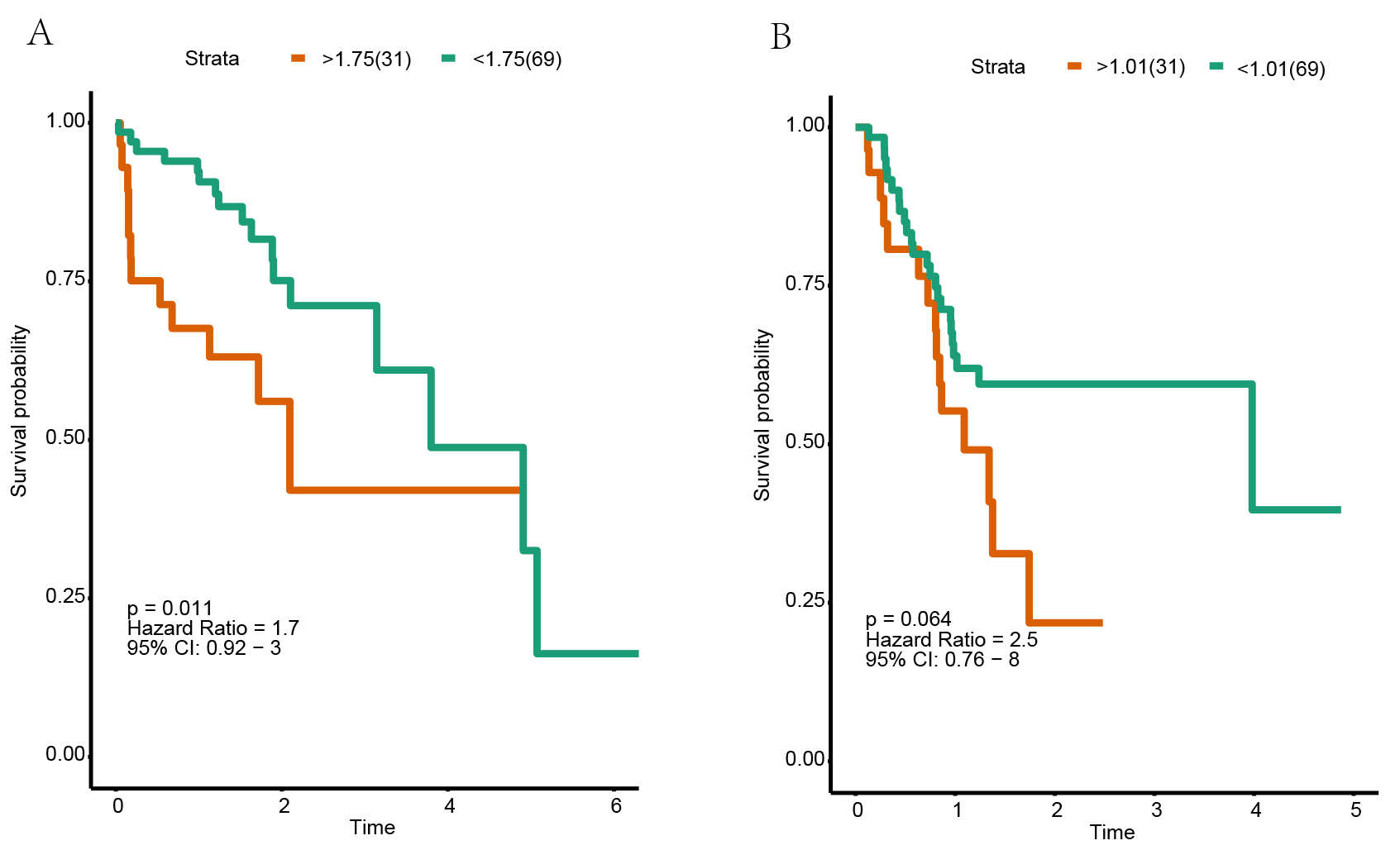

Supplement: Supplemental Material [file KBIE_A_1878763_SM2204.zip › supplement/Figure S1.tif]

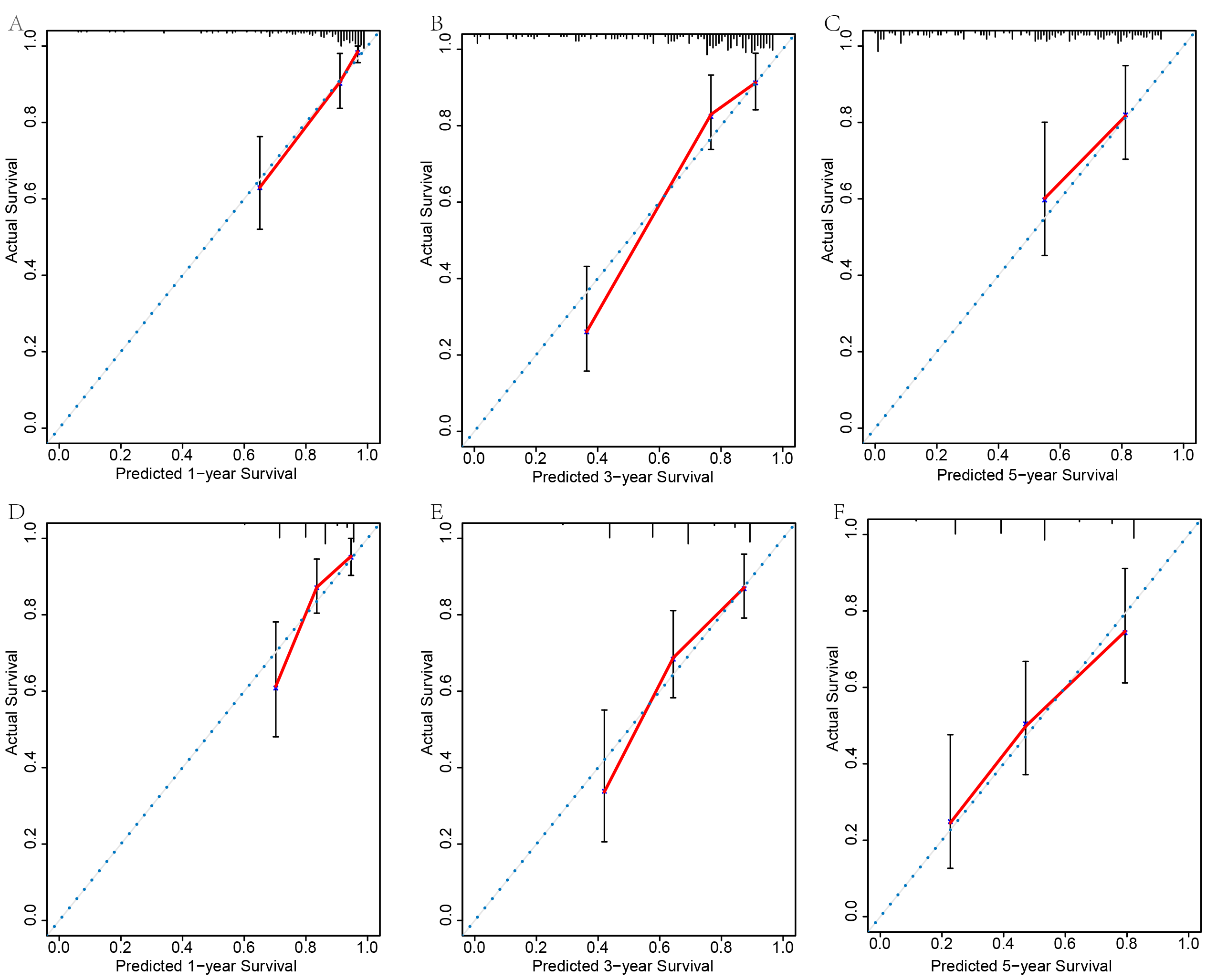

Supplement: Supplemental Material [file KBIE_A_1878763_SM2204.zip › supplement/Figure S2.tif]

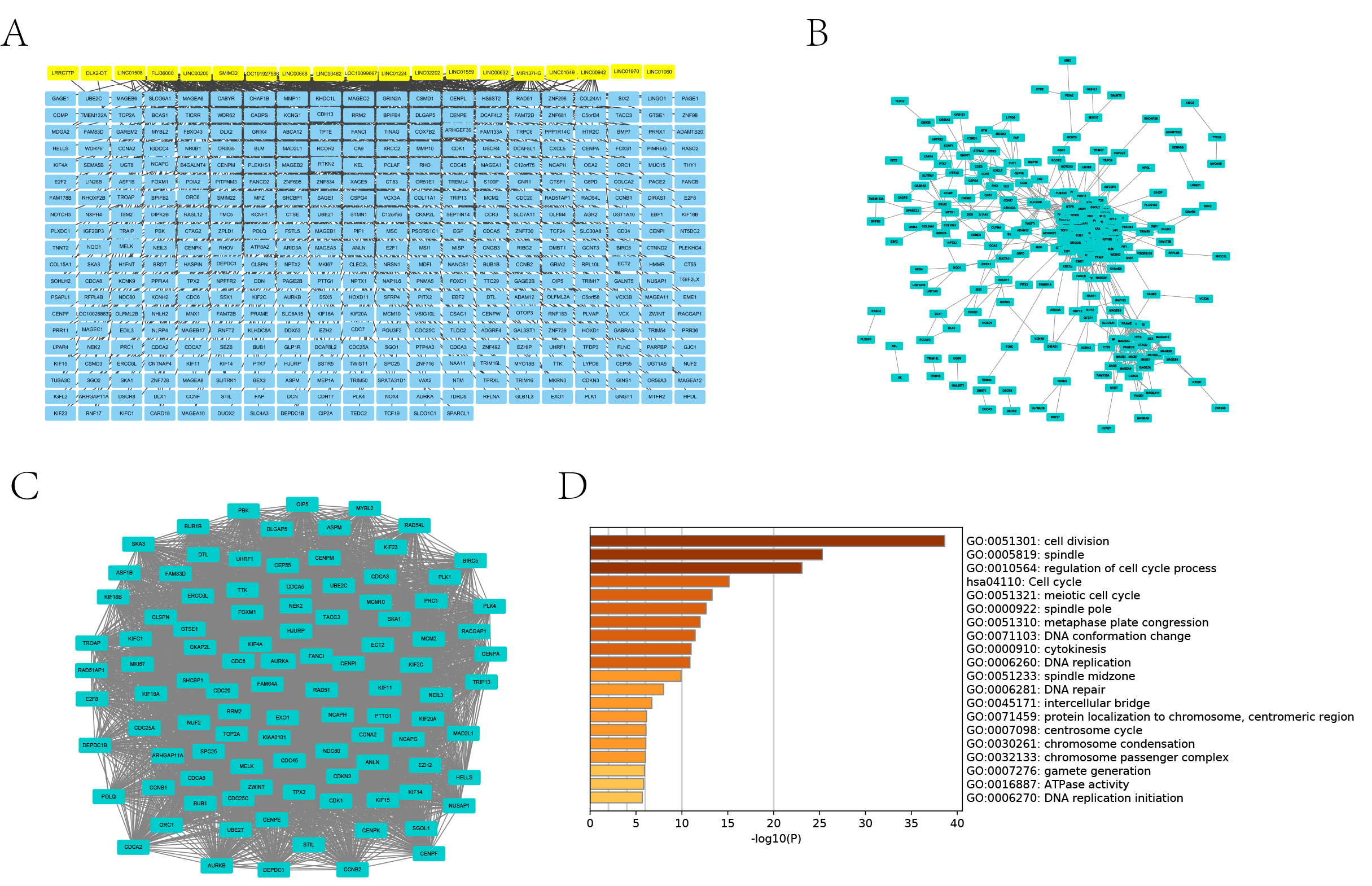

Supplement: Supplemental Material [file KBIE_A_1878763_SM2204.zip › supplement/Figure S3.tif]

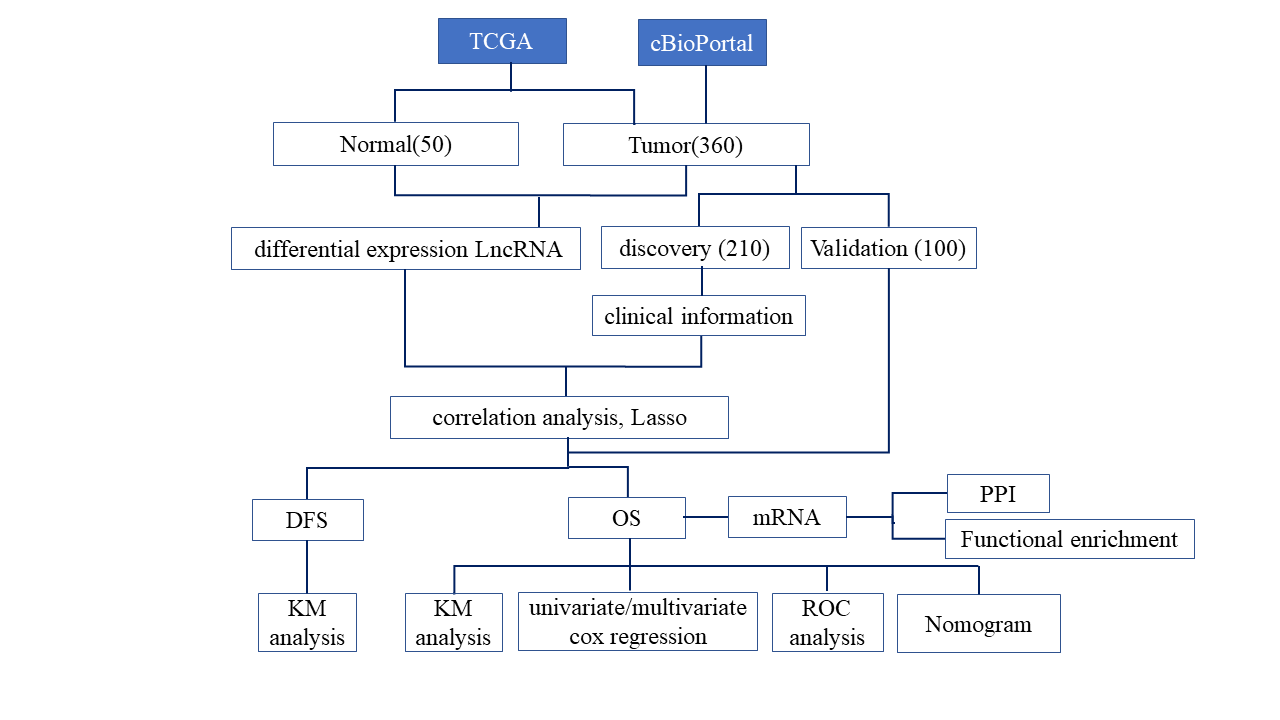

Supplement: Supplemental Material [file KBIE_A_1878763_SM2204.zip › supplement/Graphical Abstract.tif]
